# Insulin Resistance in Osteoarthritis: Similar Mechanisms to Type 2 Diabetes Mellitus

**DOI:** 10.1155/2020/4143802

**Published:** 2020-05-21

**Authors:** Elena V Tchetina, Galina A Markova, Eugeniya P Sharapova

**Affiliations:** ^1^Immunology & Molecular Biology Laboratory, Nasonova Research Institute of Rheumatology, Moscow 115522, Russia; ^2^Osteoarthritis Laboratory, Nasonova Research Institute of Rheumatology, Moscow 115522, Russia

## Abstract

Osteoarthritis (OA) and type 2 diabetes mellitus (T2D) are two of the most widespread chronic diseases. OA and T2D have common epidemiologic traits, are considered heterogenic multifactorial pathologies that develop through the interaction of genetic and environmental factors, and have common risk factors. In addition, both of these diseases often manifest in a single patient. Despite differences in clinical manifestations, both diseases are characterized by disturbances in cellular metabolism and by an insulin-resistant state primarily associated with the production and utilization of energy. However, currently, the primary cause of OA development and progression is not clear. In addition, although OA is manifested as a joint disease, evidence has accumulated that it affects the whole body. As pathological insulin resistance is viewed as a driving force of T2D development, now, we present evidence that the molecular and cellular metabolic disturbances associated with OA are linked to an insulin-resistant state similar to T2D. Moreover, the alterations in cellular energy requirements associated with insulin resistance could affect many metabolic changes in the body that eventually result in pathology and could serve as a unified mechanism that also functions in many metabolic diseases. However, these issues have not been comprehensively described. Therefore, here, we discuss the basic molecular mechanisms underlying the pathological processes associated with the development of insulin resistance; the major inducers, regulators, and metabolic consequences of insulin resistance; and instruments for controlling insulin resistance as a new approach to therapy.

## 1. Introduction

Osteoarthritis (OA) and diabetes mellitus (DM) represent the most prevalent chronic disorders. OA is prevalent primarily in women and is considered a major reason for disability and economic losses as almost half of adult OA patients are limited in their everyday activities and are restricted in their working capacity. Diabetes is associated with an increased incidence of death and serious side effects, such as stroke, chronic kidney disease, and amputation of the lower extremities [1].

OA is presently viewed as a chronic, systemic disease with slow progression and involves whole joint destruction that mainly includes degradation of hyaline articular cartilage, ligaments, menisci in the knee, hypertrophic changes of the joint capsule as well as subchondral bone remodelling, osteophyte formation, and synovial inflammation. It is accompanied by pain, dysfunction of the lower extremities, and joint deformities [2].

DM is a metabolic disorder characterized by insulin resistance (IR) and pancreatic *β*-cell dysfunction as a consequence of prolonged hyperglycaemia in the body [3]. Traditionally, DM is considered to have two forms. Type 1 diabetes mellitus (T1D) is a chronic immune-mediated disease characterized by the selective loss of insulin-producing-*β*-cells in the pancreatic islets in genetically susceptible individuals resulting in severe insulin deficiency. T1D is often observed in children and may have an autoimmune and postinfection aetiology. Type 2 diabetes mellitus (T2D) is more common in adults. T2D involves IR at the cellular level and a relative deficiency in insulin secretion. T2D development involves a progressive disturbance in glucose tolerance, which is associated with initial hyperplasia of the pancreatic islets followed by the proliferation of Langerhans *β*-cells. These processes are accompanied by an inflammatory response that induces the accumulation of the extracellular matrix (fibrosis) and the apoptotic death of islet *β*-cells, resulting in the progressive failure of *β*-cells to produce sufficient levels of insulin, followed by a decrease in insulin-stimulated glucose uptake and long-lasting hyperglycaemia. The concomitant development of osmotic and oxidative stresses produces lesions in the kidney, nervous system, and other tissues, which negatively affect the patient's lifespan [4].

OA and DM are common diseases that are significantly associated. Epidemiological studies report that the overall risk of OA in patients with DM is 1.46 while that of DM in patients with OA is 1.41 [5]. The prevalence of OA among patients with DM and that of DM in patients with OA was 29.5% and 14.4%, respectively [5]. Additionally, T2D independently predicts the risk of total joint replacement [6] and is considered a specific risk factor for OA. T2D related hyperglycaemia and IR result in loss of insulin receptor sensitivity, which might reduce chondrocyte survival and differentiation capacity, thereby accelerating OA progression [7]. Manifestations of OA are commonly observed in patients with T1D who lack the ability to produce insulin [8]. For example, radiological glenohumeral OA was observed in 35% T1D patients [9]. This is also associated with hyperglycaemia, which occurs in patients with both types of DM, and DM is known to negatively affect connective tissues [10].

Studies have reported a stronger association between TD and knee OA in women; however, no such association was observed in women aged <30 years [11], implicating the role of oestrogen in the pathogenesis of both conditions. Animal studies have shown that ovariectomy increased the risk of T2D [12]; in contrast, a few studies have shown that 17*β*-estradiol (ovarian oestrogen) improves insulin sensitivity and protects insulin production in the diabetic state [13]. Mechanistically, glucose-stimulated insulin secretion requires an increase in the intracellular calcium concentration that is facilitated by oestrogen. This process involves an increase in the adenosine triphosphate (ATP):adenosine diphosphate (ADP) ratio in pancreatic *β*-cells, closure of ATP-sensitive potassium channels, depolarization of cell membrane, and opening of L-type voltage-dependent calcium channels [14].

Furthermore, OA and T2D are significantly associated with obesity. For example, a positive association is observed between both overweight and obesity and the risk of hand, hip, and knee OA. The strength of this association increases in a dose-dependent manner with increasing body mass index (BMI). For example, incidence rates of knee OA were 5-fold higher in patients with grade II obese than in individuals at a normal weight [15]. Recent studies have reported a significant association between BMI and T2D (relative risk = 6.88) [16]. These effects are attributable to the release of increased quantities of proinflammatory mediators, such as tumour necrosis factor (TNF)*α*, interleukin (IL)-6, and IL-1 from adipocytes in individuals with obesity. These agents are known to trigger systemic low-grade inflammation in patients with both OA and TD [17].

Therefore, T2D and OA are both associated with patient age, low-grade inflammation, and obesity and result in disturbances in cellular metabolism; these disorders might have common pathophysiological mechanisms [1]. These similarities suggest that common approaches could be applied to ameliorate the metabolic imbalance associated with these diseases. Therefore, the aim of the study was to demonstrate that OA is not a disease solely of a single joint but like T2D is a pathology of the whole body, which results from insulin resistance-related traits that develop before the onset of the disease. Besides, we suggest that common mechanisms in the development of metabolic disturbances related to both pathologies might be associated with an imbalance in energy provision. These issues will be discussed in the present review.

## 2. Methodology

A comprehensive search of peer-reviewed literature published in the past two decades was undertaken. The search was performed in Medline/PubMed using the following keywords: (“osteoarthritis” OR “articular cartilage” OR “type 2 diabetes mellitus”) AND (“inflammation” OR “obesity” OR “oxidative phosphorylation” OR “AMPK” OR “insulin resistance” OR “fibrosis” OR “ATP” OR “exercise” OR “pain”). Exclusion criteria were as follows: studies not centred on the outcome of interest; studies only with abstract or with no available data; studies where a full description of research design was not provided or available; papers not written in English; all grey literature; duplication publications; educational materials and research publications intended for health care professionals to help them give verbal recommendations to patients; theses, forecasts, and journals not accessible online; and PowerPoint presentations, blogs, or websites. In addition, the reference lists of appropriate studies were also examined to identify additional relevant studies. Both authors independently screened all titles and abstracts for eligible studies and extracted data independently. Full texts for all relevant papers were collected and reevaluated by both authors for appropriateness. All papers satisfying the criteria and approved by both authors were included. No search restrictions were imposed.

## 3. Results and Discussion

### 3.1. Insulin Is a Universal Regulator of Cellular Metabolism

Insulin controls the expression and activity of more than 150 genes in many organs and tissues by regulating their transcription, messenger ribonucleic acid (mRNA) stability, and translation [18]. Mechanistically, the binding of insulin to the insulin receptor results in the autophosphorylation of its tyrosine residues. Receptor activation leads to the phosphorylation of tyrosine residues on adaptor proteins, members of the insulin receptor substrate family (IRS), which promote signalling to downstream targets [19].

Insulin is primarily involved in the maintenance of physiological blood glucose levels which depends on a complex interaction between the insulin responsiveness of skeletal muscle, liver, and adipose tissue and glucose-stimulated insulin secretion by pancreatic *β*-cells [20]. For example, in adipose cells, glucose transporters (GLUTs) are located intracellularly in the absence of insulin. Insulin stimulates the phosphorylation of tyrosine residues on IRS-1 and allows insulin receptors to bind to GLUT, subsequently inducing the recruitment of GLUT from the extracellular pool to the plasma membrane and exposing functional glucose transporters to the extracellular compartment containing glucose [21].

In pancreatic *β*-cells, respiration and the rate of adenosine triphosphate (ATP) production in oxidative phosphorylation (OXPHOS) are highly dependent on glucose availability due to the inability of *β*-cells to metabolize glucose by aerobic glycolysis, as *β*-cells are not capable of converting pyruvate to lactate [22]. Therefore, when the glucose concentration in circulation increases after a meal, the increased ATP/ADP (adenosine diphosphate) ratio in *β*-cells initiates the closing of ATP-sensitive K-channels, thus depolarizing the plasma membrane and activating voltage-gated L-type Ca2^+^ channels to stimulate Ca2^+^-dependent exocytosis of insulin-containing secretory granules [23]. As in the majority of other cell types, cellular energy requirements are regulated by respiration rates and ATP availability; the increase in circulating insulin concentrations initiates biosynthetic activity in different tissues such as skeletal muscle, liver, white adipose tissue, and brain for the utilization of increased nutrient supply followed by restoration of the blood glucose concentration to its original level [24].

Insulin is also of primary importance for articular cartilage. Human chondrocytes express the functional insulin receptor, which is more abundant in healthy cells relative to OA. Insulin is capable of inducing anabolic responses that promote the synthesis of type II collagen and proteoglycan, inhibit expression of the collagenases, matrix metalloproteinase- (MMP-) 1 and 13, and a disintegrin and metalloproteinase with thrombospondin motifs (ADAMTS), and counteract the effects related to IL-1*β* [25]. In animal and human studies, insulin treatment reversed cartilage loss, which was associated with fracture healing in diabetic mice [26] and ameliorated cartilage degradation [27].

### 3.2. Mechanisms of Insulin Resistance Development

The IR state involves induction of the inhibitory serine phosphorylation of IRS-1, preventing the involvement of IRS-1 in insulin receptor signalling and thus inhibiting insulin action [19]. In an insulin-resistant state, the ability of insulin to stimulate glucose uptake via insulin-dependent GLUTs is impaired, requiring higher than normal concentrations of extracellular insulin to maintain normal circulating glucose levels. Initially, higher insulin concentrations are maintained by *β*-cell overstimulation. Persistent insulin activation induces hyperphosphorylation of the serine/threonine (Ser/Thr) residues on the IRS. This hyperphosphorylation results in reduced insulin sensitivity of the insulin receptor, inhibition of insulin signalling, or the uncoupling of IRS-1 and its degradation [28].

### 3.3. Physiological Insulin Resistance

Physiological insulin resistance in healthy cells represents a survival strategy and resolves after cessation of the abnormal condition. The survival of every organism depends on the capacity to withstand starvation and develop a strong acute immune response to pathogens or tissue injury [29]. For this purpose, the energy obtained from nutrients should be transferred for expenditure during a strong immune response. The inflammatory response involves proinflammatory cytokine production, which is an energy-consuming process [30]. Proinflammatory cells rely on aerobic glycolysis to convert pyruvate to lactate, and in an alternative route, reoxidising the NADH produced by glycolysis. Simultaneously, these cells downregulate oxidative mitochondrial metabolism, which has an anti-inflammatory effect. This downregulation is accompanied by changes in the tricarboxylic acid (TCA) cycle function from a catabolic function that generates ATP into a partially anabolic function, as citrate withdrawal from the cycle is used for fatty acid synthesis [30].

As adipocytes and hepatocytes are located adjacent to blood vessels carrying immune cells (macrophages), they would be the first cells to interact with the immune system. Therefore, the activation of the inflammatory response would transiently block major anabolic signalling pathways such as the insulin pathway in tissues other than immune cells by activating serine kinases such as c-Jun N-terminal kinase (JNK), I*κ*B kinase (IKK)*β*, extracellular-signal-regulated kinase (ERK), ribosomal protein S6 kinase (S6k), mechanistic target of rapamycin (mTOR), protein kinase C (PKC), and glycogen synthase kinase (GSK)3*β* and inducing inhibitory serine phosphorylation of IRS-1 [31]. Therefore, inflammation serves as a driver of physiological IR, which is associated with transient redirection of energy for immune system activation.

### 3.4. Pathological Insulin Resistance

The insulin-resistant state is an early and key feature of T2D that develops 10–20 years before the onset of the disease [11]. Recently, IR traits were also described in OA patients [32]. For example, an increase in serum insulin concentration was found in 82% of patients with OA, while high insulin concentrations (10–500 nM) were shown to be capable of increasing articular cartilage degradation [33].

#### 3.4.1. Overnutrition

**Figure 1:**
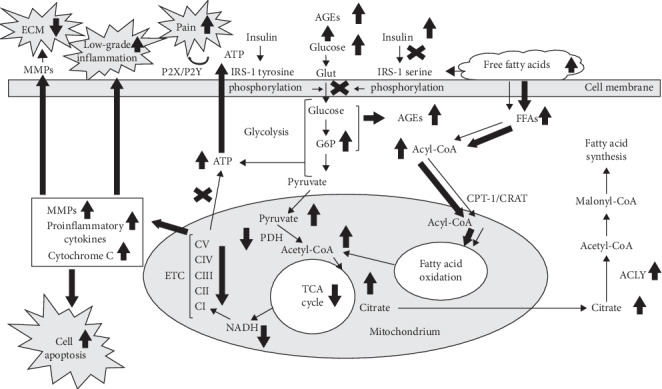
Metabolic changes in healthy and osteoarthritic chondrocytes associated with insulin resistance. Thin arrows, healthy chondrocyte. Bold arrows, insulin resistance-related changes in osteoarthritic chondrocyte. ECM, extracellular matrix; MMPs-matrix metalloproteinases; P2X/P2Y, purinergic receptors; IRS, insulin receptor substrate; ATP, adenosine triphosphate; PDH, pyruvate dehydrogenase; ETC, electron transport chain; С, Complex; NADH, nicotinamide adenine dinucleotide reduced; G6P, glucose-6-phosphate; AGEs, advanced glycation endproducts; TCA, tricarboxylic acid; FFAs, free fatty acids; CPT, carnitine:palmitoyl-CoA transferase; CRAT, carnitine acyltransferase; ACLY, ATP-citrate lyase.

Pathological insulin resistance in OA patients might result from overnutrition (Figure 1). According to the obesity-related model of IR development, increased oxidation of fatty acids (FAs) produces high levels of intracellular acetyl-CoA and citrate, which are capable of inhibiting two enzymes involved in glucose utilization, namely, pyruvate dehydrogenase and phosphofructokinase, a key rate-limiting enzyme in glycolysis. Subsequent accumulation of glucose-6-phosphate would inhibit hexokinase II activity, resulting in an increase in intracellular glucose concentrations and decreased glucose uptake [34].

It was also shown that fatty acids could affect the GLUT4 transporter directly while elevated plasma FA concentrations abolished insulin-stimulated IRS-associated phosphoinositide 3- (PI3-) kinase activity by phosphorylation of serine/threonine sites on insulin receptor substrates, which fail to associate with PI3-kinase and thus decreased activation of glucose transport. Therefore, the accumulation of intracellular fatty-acyl-CoAs or other FA metabolites leads to insulin signalling defects and IR [35].

A sustained high citrate concentration associated with IR could also result from the low expression of ATP-citrate lyase (ACLY), which was observed in the pancreatic *β*-cells of obese patients [36]. ACLY is a cytosolic protein that catalyses the formation of acetyl-CoA and oxaloacetate from citrate and coenzyme A in the presence of ATP and thus regulates the switch between glucose and fatty acid metabolism.

The increase in glycolytic flux can be associated with a switch in the expression of liver phosphofructokinase-2/fructose-2,6-bisphosphatase (PFKFB) isoforms from PFKFB1 to PFKFB3 and results in the accumulation of fructose-2,6-bisphosphate [30]. Indeed, PFKFB3 disruption in mice ameliorated high-fat diet-induced adipose tissue inflammation but exacerbated systemic IR, whereas the overexpression of PFKFB3 in adipose tissue improved systemic insulin sensitivity [37].

Obesity due to overnutrition likewise affects the severity of joint degradation, risk of OA progression, and joint surgery requirements [38]. At the same time, significant weight loss was required to achieve symptomatic and structural improvements in humans, while moderate weight loss protected subjects at high risk of knee OA [39]. The downregulation of PFKFB3 expression and increased activity of citrate synthase were also observed in human OA cartilage and in TNF*α*- or IL-1*β*-treated chondrocytes [40].

Adipokines, primarily resistin, are shown to be involved in the development of IR in patients with DM and OA [41]. Resistin is a 12 kDa cysteine-rich polypeptide hormone secreted by macrophages and adipocytes. It suppresses insulin-induced glucose uptake [42], promotes the proliferation, migration, and activation of human endothelial cells, increases expression of vascular endothelial growth factor receptors and MMPs, and activates ERK1/2 and p38 signalling pathways [43]. Recent studies have reported that insulin function regulation is initiated in hypothalamic nuclei, which control energy homeostasis [44], and resistin reduces insulin sensitivity and signalling in the hypothalamic nuclei, as well as in adipose tissue, liver, and skeletal muscles by increasing serine phosphorylation of insulin receptor substrate-1 (IRS1) [41].

Resistin was also associated with radiographic knee OA, promoted OA progression, was positively correlated with OA severity and leptin levels, and induced production of MMPs and proinflammatory cytokines in human chondrocytes [45]. Interestingly, resistin was not associated with cartilage volume loss and even promoted cartilage proteoglycan synthesis [46]. Therefore, the adverse effects of resistin in patients with OA might be attributable to increased expression of proinflammatory cytokines, as well as the production of tissue degrading enzymes and downregulation of synthesis of structural proteins, which are formed in the pericellular matrix of the articular cartilage [47].

#### 3.4.2. Inflammation

Increased nutrient concentrations associated with long-term overnutrition can induce inflammation in all tissues involved in energy homeostasis, such as fat, muscle, liver, islets, and nutrient-transporting blood vessels [29]. Free fatty acids (FFAs) are considered to be important proinflammatory agents that can activate the innate immune system. For example, FFAs can either bind directly to Toll-like receptors (TLRs) or induce the TLR dimerization required for signalling [48]. Adaptive immunity can also be involved in IR development, promoting the release of autoantigens in concert with alarmins and further activating the immune system or stimulating obesity-induced inflammation due to the accumulation of T cells in adipose tissue. This mechanism is supported by the elevated circulating levels of acute-phase proteins, cytokines, and chemokines observed in T2D and OA patients. In addition, proinflammatory cytokines such as TNF*α* are capable of activating various signal transduction pathways that inhibit insulin action and promote hyperglycaemia [49].

Obesity also triggers a local low-grade subacute inflammatory response due to the infiltration of proinflammatory M1-polarized macrophages in OA synovium prior to cartilage degradation [50]. These findings are supported by the observation that, in early OA, 266 genes were differentially expressed between inflamed synovium and healthy synovial tissue [51]. Furthermore, increased concentrations of IL-1*α*, IL-18, and TNF*α* were associated with OA severity, while baseline IL-18 levels predicted OA progression [32]. Besides, patients with knee OA had increased the levels of IL-6 released from the infrapatellar fat pad relative to subcutaneous adipose tissue while individuals with a higher baseline body mass index and increased serum IL-6 levels were more likely to be diagnosed with radiographic knee OA after a 5-year follow-up [52].

Therefore, pathological IR results from disturbances of cellular metabolic processes, primarily triggered by overnutrition, and is associated with chronic low-grade inflammation.

#### 3.4.3. Hyperglycaemia

In many cells and tissues, primarily articular chondrocytes, glucose serves as a major energy-producing substrate. During pathological IR conditions (Figure 1), the above-described inhibition of the glycolytic pathway produces an increase in intracellular glucose and glucose-6-phosphate concentrations, potentially resulting in reduced insulin-stimulated glucose transport activity in target tissues [25]. Subsequent transient upregulation of glycolysis increases intracellular ATP concentrations as indicated by functional inhibition of K(ATP) channels, which sense intracellular ATP/ADP levels and are impaired in OA chondrocytes [53].

Glucose and glucose-6-phosphate accumulation can result in the formation of advanced glycation end products (AGEs), which are formed by the nonenzymatic glycation of reducing sugars such as glucose, with amino groups of proteins, lipids, and nucleic acids [54]. Glycation is a concentration-dependent process that is enhanced in diabetes and aging and was observed in OA [55]. AGE-related inhibition of ATP production and AMP-activated protein kinase (AMPK) phosphorylation [56] further point to high ATP concentrations in OA chondrocytes. In addition, AGEs can induce transcription factor nuclear factor kappa-light-chain-enhancer of activated B cells (NF-*κ*B) and stress kinase ERK1/2 expression through the activation of the recognition receptor RAGE, followed by NOD-like receptor (NLR) family member NLRP3 inflammasome formation, and IL-1 receptor activation and thereby systemic inflammation [57].

#### 3.4.4. Mitochondrial Dysfunction

Disturbances in mitochondrial FA oxidation associated with pathological IR might result from carnitine insufficiency and/or carnitine acetyltransferase (CRAT) deficiency (Figure 1). Carnitine is responsible for mitochondrial transportation and oxidation of long-chain FAs and also serves as an acceptor of acyl groups, which enables the export of excess of acylcarnitines from mitochondria. Carnitine requirements are increased during sustained metabolic stress, while decreased carnitine availability is associated with IR states such as old age, diabetes, and diet-induced obesity [58]. In human knee OA, L-carnitine supplementation improved the patient's clinical status, demonstrating significant reductions in serum malondialdehyde, total cholesterol, and low-density lipoprotein cholesterol concentrations compared to those at baseline. This was accompanied by a significant decrease in serum IL-1*β* and MMP-1 levels [59].

Another possibility regarding the development of IR-related mitochondrial dysfunction might involve insufficient cellular antioxidant activity. The mitochondrial respiratory chain is the main source of superoxide radicals in many cell types, including pancreatic *β*-cells [60]. 0.15% of total oxygen used in OXPHOS is converted to superoxide anion, which is transformed to hydrogen peroxide by superoxide dismutase (SOD) [61]. Superoxide production is increased in *β*-cells in rodents with T2D, in cultured *β*-cells under hyperglycaemic conditions or lipid excess in obese humans, and is associated with attenuation of the respiratory chain [62]. However, SOD activity was reduced in T2D patients [63], in human OA chondrocytes and articular cartilage [64]. Reduced activities of Complexes I, II, and III of the mitochondrial respiratory chain and mitochondrial depolarization in human OA articular chondrocytes in culture relative to normal chondrocytes were also noted, while the inhibition of Complexes III and V were capable of stimulating proinflammatory cytokine and matrix metalloproteinase production in these cells [65].

Overall, the attenuation of mitochondrial function during IR development is linked to the reduction of mitochondrial oxidative enzyme and electron transport chain activity and hence decreased ATP synthesis.

#### 3.4.5. Insulin Resistance-Related Pain

IR might also be associated with pain manifestations involving central sensitization [66]. It has been shown that, in addition to peripheral tissues, the central nervous system is also involved in the regulation of tissue-specific insulin sensitivity, as insulin receptors were found throughout the brain [67]. The origin of insulin in the brain is mostly peripheral, as insulin crosses the blood brain barrier by insulin receptor-mediated active transport [68]. Studies in diabetic rats have demonstrated that nerve injury is capable of downregulating the expression of insulin receptors in skeletal muscle innervated by the injured nerve [69] while acute pain has been reported to reduce insulin sensitivity due to decreased glucose uptake in the body [70].

Another mechanism of IR involvement in the increased pain sensitivity in OA and T2D might be associated with excessive amounts of cellular ATP, a key pain mediator capable of activating purinergic receptors P2X and P2Y, which are involved in both neuropathic diabetic and rheumatic pain sensitivity and hyperalgesia in arthritic joints [71] (Figure 1). For example, extracellular ATP was detected in most nonexcitatory cells, including chondrocytes, presumably due to nonlytic ATP release by conductance effluxes, specific transporters, constitutive secretory pathways, or regulated exocytosis [72]. This is supported by the observation that increased ATP levels were present in the knee synovial fluid of OA patients and were related to pain intensity [73].

### 3.5. AMPK Is a Major Regulator of Insulin Resistance

AMPK is a serine/threonine kinase that regulates metabolic pathways responsible for cellular energy generation and is involved in the control of whole-body energy balance by responding to the hormonal and nutrient signals in the central nervous system and peripheral tissues that govern nutrient uptake and energy expenditure [30]. AMPK is activated by adenosine monophosphate (AMP) and antagonized by increased concentrations of ATP, thus controlling changes in the AMP/ATP ratio. AMPK stimulated glucose uptake and suppressed glycogen synthesis in skeletal muscle [74]. AMPK activation inhibits anabolic pathways and upregulates catabolic pathways.

The major AMPK-regulated pathway, which reduces the risk of obesity and IR, stimulates fatty acid (FA) oxidation and inhibits FA synthesis by phosphorylating and downregulating acetyl-CoA carboxylase, a rate-limiting reaction in the conversion of acetyl-CoA to malonyl-CoA. In addition, AMPK stimulates malonyl-CoA decarboxylase, which catalyses malonyl-CoA degradation. A decrease in malonyl-CoA prevents the inhibition of carnitine:palmitoyl-CoA transferase-1 (CPT-1), which is responsible for long-chain fatty acyl-CoA transport into mitochondria and fatty acid synthase controlling FA biosynthesis [75]. Chronic AMPK activation has been shown to stimulate mitochondrial biogenesis in skeletal muscle mediated by an increase in the concentration of proliferator-activated receptor *γ* coactivator- (PGC-) 1*α*, which is downregulated in T2D and OA [76, 77]. AMPK can increase insulin sensitivity either by suppressing the mechanistic target of rapamycin (mTOR) directly or by activating its inhibitor tuberous sclerosis complex (TSC), resulting in decreased activity of the TSC-mTOR-S6K1 pathway [78].

#### 3.5.1. AMPK as an Indicator of the Efficacy of ATP Turnover

Effective oxidative capacity is determined by energy expenditure, and therefore, the mitochondrial respiratory dysfunction associated with elevated FFA levels reflects decreased ATP turnover [79]. For example, the treatment of primary myoblasts with palmitate was associated with a decreased rate of de novo protein synthesis, a major ATP consumer. Generally, FFAs reduce the overall absolute rate of mitochondrial ATP supply, which may be a consequence of lower ATP demand. Therefore, depressed mitochondrial respiration followed by decreased mitochondrial ATP synthesis might not involve intrinsic defects but could be an adaptive response to altered energy demand [80]. In addition, the treatment of cultured bovine articular chondrocytes with high doses of exogenous ATP induced an increase in extracellular inorganic phosphate accumulation, a by-product of ATP degradation. This effect was accompanied by the upregulation of MMP-13 and proinflammatory mediators, preventing extracellular matrix (ECM) formation [81].

#### 3.5.2. AMPK Activity and Insulin Resistance

High insulin concentrations inhibit AMPK [82]. Animal studies using transgenic mice that expressed the inactive form of the AMPK*α*2 subunit in skeletal muscle developed impaired whole-body glucose tolerance and IR in skeletal muscle, specifically when fed a high-fat diet [83]. These results were supported by genetic studies in the Japanese population, which demonstrated that polymorphisms in the gene encoding the AMPK*α*2 subunit were linked to IR and T2D [84]. The decrease in AMPK activity associated with the IR state has been also shown in the adipose tissue of 75% of severely obese patients undergoing bariatric surgery. This association was accompanied by an upregulation of proinflammatory genes, including cytokines and chemokines, and the decreased expression of PGC-1*α*, enzymes related to the *β*-oxidation of FA and the citric acid cycle in visceral adipose tissue [85]. Skeletal muscle AMPK expression and activity were also decreased in T2D patients [86].

Phosphorylated AMPK*α* is constitutively present in normal articular chondrocytes and cartilage, while OA chondrocytes are deficient in AMPK activity [87]. Decreased AMPK activity enhances the procatabolic responses of chondrocytes to the proinflammatory cytokines IL-1*β* and TNF*α* [88]. Alternatively, the upregulation of AMPK was associated with decreased collagen degradation activity and increased the expression of type II collagen accompanied by the upregulation of TCA-related genes and the downregulation of proinflammatory cytokines and metalloproteinases [89]. In animal studies, pharmacological AMPK activation limited knee OA development and progression [90].

Therefore, AMPK is a primary regulator of cellular energy metabolism and IR state, while the acquisition of AMPK expression levels similar to that in healthy subjects could serve as an indicator of treatment efficacy.

### 3.6. Putative Role of Chronic Inflammation and Fibrosis in Energy Balance Improvement

Physiological inflammation represents a short-term adaptive response as a component of tissue repair and involves the integration of numerous signals in different cells and organs [29]. For example, the activation of NFkB observed in pregnancy is mediated by limited hyperglycaemia that induces *β*-cell proliferation and increase in the pancreas mass pointing to the increased requirements for insulin [91].

The prolonged, low-grade or chronic inflammation observed in many metabolic diseases including OA and T2D is generally not considered beneficial. Chronic inflammation is associated with the proliferation of immune cells, abnormal production of cytokines, and activation of proinflammatory signalling pathways [31]. Chronic inflammation was observed in T2D patients, while increases in interleukin- (IL-) 1*β*, IL-6, and C-reactive protein (CRP) levels were considered predictors of the disease [92]. Differences in cytokine/chemokine levels were also found between normal and OA serum samples [93]. Increased expression of IL-1*β* and TNF*α* was observed in the peripheral blood of OA patients relative to that of healthy controls [94]. In addition, knee cartilage volume was negatively associated with the concentrations of the circulating cytokines IL-6 and TNF*α* and CRP [95].

Furthermore, chronic inflammation is associated with tissue fibrosis, which is characterized by fibroblast proliferation and excessive ECM deposition [96]. For example, high glucose levels stimulated TLR4 and resulted in NF-*κ*B activation followed by upregulation of growth factors such as transforming growth factor (TGF)*β* and connective tissue growth factor (CTGF), which was accompanied by ECM accumulation in tendons in T2D patients [97]. These effects were associated with the enhanced expression of MMP-1, MMP-2, and MMP-9, reflecting the increase in ECM degradation under hyperglycaemic conditions [98]. In human OA, inflammation and fibrosis also occur together with ECM destruction, as early changes prior to radiographic alterations were related to synovial inflammation, indicated by thickening of the lining layer and an increase in the proliferation of lining cells in sites of chondral defects [99] and by profibrotic changes in infrapatellar fat pad cells [100].

However, protein synthesis and cell proliferation are highly ATP-dependent energy-consuming processes [101]. The reduced AMPK expression in OA articular chondrocytes [88, 89] and in the cells of IR tissues in T2D [86] suggests increased levels of intracellular ATP. Additionally, the chronic systemic inflammation associated with profibrotic changes in tissues is accompanied by increased AMPK expression in peripheral blood, indicating additional energy requirements [89]. These effects might induce an energy appeal reaction, a process that involves the redirection of energy-rich fuels from energy-rich compartments, such as IR tissues in T2D and OA articular chondrocytes, as has been indicated by studies of the activated immune system [102].

Therefore, as catabolic pathways are responsible for energy provision [103], we may speculate that the redirection of energy fuels to support immune cell functions and/or fibrotic tissue synthesis might improve the energy balance of the whole body, which is disturbed by the disease.

### 3.7. Factors Associated with Control of Insulin Resistance in Osteoarthritis

OA is considered an incurable disease; therefore, all current treatments are symptomatic in nature and are primarily focused on managing the secondary manifestations of IR-induced profound disturbances of cellular energy metabolism such as inflammation, articular cartilage destruction, and pain. Notably, OA is treated with paracetamol, nonsteroidal anti-inflammatory drugs (NSAIDs), corticosteroid injections, and tramadol based on Guidelines [104]. Novel treatments such as capsaicin (a transient receptor potential cation channel subfamily V member 1 (TPRV1) agonist) and galcanezumab (for calcitonin gene-related peptide (CGRP) blockade) target pain, whereas biological agents such as lutikizumab (for IL-1*α*/*β* blockade) or etanercept (for TNF blockade) target inflammation [105]. However, some of these agents are associated with prodiabetic effects. For example, glucosamine (the most widely used OA medication in postmenopausal women) is known to cause glucose intolerance and IR and inhibits insulin production by pancreatic *β*-cells, resulting in apoptosis and deranged glucose metabolism [106]. Therefore, research is focused on the development of newer approaches to target pathways affected by the OA.

#### 3.7.1. Cellular and Subcellular Particle-Based Therapies

Cell therapies utilizing stem, progenitor, primary, or stem cell derivatives (including embryonic stem cells, induced pluripotent stem cells, or mesenchymal stem cells (MSCs)) constitute a promising therapeutic approach to OA. These cells replace injured cells or tissues to stimulate the self-healing of endogenous tissues through nutritive effects [107]. However, studies have shown that MSCs promote carcinogenesis and maldifferentiation of cells into types that may cause disease exacerbation [108]. Therefore, recently extracellular vesicles (exosomes) that are actively secreted by MSCs are considered important contributors for OA amelioration [109]. Exosomes are nanosized membranous vesicles (30–120 nm in diameter), which are released by many cell types [110] and are involved in the intercellular transfer of bioactive molecules such as proteins, lipids, mRNAs, or microRNAs. Exosomes respond to cell injury and participate in the regulation of cell proliferation and differentiation [111] and may contribute to cartilage repair and regeneration [112]. MSC exosomes were shown to alleviate pain, inflammation, and matrix degradation via activation of the ERK, protein kinase B (Akt), and AMPK pathways in an animal model of temporomandibular OA [113]. Exosomes have also shown efficacy as potential therapeutic agents for T1D in animal studies [114]. The absence of genomic content and the consequent lack of replication capacity reduce the malignant potential of exosomes [115]. However, the widespread application of exosomes is limited by the lack of adequate knowledge regarding the exact molecular mechanisms through which they participate in cartilage repair [109]. For example, some studies have reported that chondrocytes showed decreased anabolic activity in response to MSC exosomes unless they were capable of expressing miR-140-5p [116]. Additionally, exosomes from different cell types are highly heterogeneous and show varying survival and healing capacities secondary to old age or unfavourable health conditions in donors [117].

The recent introduction of high-throughput sequencing has resulted in the identification of endogenous circular RNAs (circRNAs) derived from precursor mRNA in different tissues [118]. CircRNAs represent endogenous noncoding RNAs characterized by covalently closed cyclic structures with nonpolyadenylated tails, which function as translational regulators, RNA-binding sponges, and microRNA sponges [119]. They are widely expressed, highly stable, evolutionary conserved elements among species that show tissue-specific and developmental stage-specific expression patterns [120]. For example, research has shown that some circRNAs were aberrantly expressed in articular cartilage of patients with OA and underwent upregulation in chondrocytes stimulated by IL-1 and TNF*α* [121]. Overexpression of some circRNAs was associated with the upregulation of ECM degrading enzymes and inhibition of matrix proteins such as type II collagen and aggrecan [122]. Dysregulation of circRNA was also observed in patients with DM and was primarily associated with regulation of insulin secretion by *β*-cells as well as vascular complications including diabetic nephropathy, neuropathy, and cardiomyopathy [118]. CircRNAs are also involved in the regulation of miRNA activity by the formation of miRNA sponges that interact with RNA-binding proteins (RBPs) to regulate gene transcription and protein translation [123]. Notably, miRNAs represent small noncoding RNAs capable of posttranslational regulation of target mRNAs transcription via binding the corresponding miRNA response elements (MREs) [124]. Some circRNAs contain MREs that compete for miRNA-binding sites (miRNA sponges) and result in functional sequestration of miRNAs [125]. Therefore, circRNAs are considered useful diagnostic and predictive biomarkers of some disorders, and inhibition of their activity might promote ECM recovery and slow down the process of joint degradation [123]. It is noteworthy that although many circRNAs are described in humans, only a few are known to produce biological effects [126]. Other studies investigating circRNA silencing by specific siRNAs have reported a lack of stability against extracellular and intracellular nucleases and a number of nonspecific effects [127].

#### 3.7.2. Therapeutic Potential of Mimetic Molecules

Recent in vitro and animal studies have suggested that OA could be ameliorated using mimetics of known molecules affected by the disease. For example, lubricin mimetic, an extracellular matrix component structural analogue, attenuated OA progression [128] whereas PG545, a heparan sulphate mimetic in cultured human OA chondrocytes, inhibited the effect of heparanase that is known to stimulate catabolic MMP13 and ADAMTS and downregulate aggrecan and type II collagen synthesis [129]. Animal studies have proved that the glucose tolerance factor (GTF), a dietary agent extracted from yeast, functions as a putative insulin mimetic to lower blood glucose and lipid concentrations. It is also known to participate in phosphorylation of IRS1, Akt, and mitogen-activated protein kinase (MAPK) independent of insulin receptor phosphorylation [130]. However, these effects are questionable because GTF has not been purified, and its chemical structure remains unclear. Cobalt chloride, a hypoxia-mimetic agent has been used to confirm the role of glucose metabolism in articular chondrocytes. These studies have shown that the energy metabolism of chondrocytes undergoes adaptations under hypoxic conditions; however, reduced GLUT1 activity impairs OA cartilage repair [131]. Synthetic SOD mimetics such as nitroxides (tempol) and compound M40403 efficiently decreased hydroxyl radical production and tissue inflammation and injury and also reduced neutrophil infiltration at the site of inflammation in addition to protecting against cell death and loss of mitochondrial content in articular cartilage after mechanical overload [132].

Furthermore, studies on mimetic molecules might unravel some molecular mechanisms of the disease. For example, the analysis of SOD mimetics has shown upregulation of superoxide production only during normal articular chondrocyte loading, whereas chronic overloading resulted in more complex changes involving altered lipid metabolism [133]. This finding might be attributed to the fact that anabolic responses of the articular cartilage to normal loads depend on mitochondrial electron transport and, therefore, the mitochondrial function can control chondrocyte inflammatory response [134] evidenced by attenuation of such a response following the inhibition of electron transport by rotenone (mitochondrial Complex I inhibitor) or by the addition of antioxidants [135]. In contrast, severe impact significantly increases oxidant production, and prevention of cell death could be achieved only by antioxidant supplementation or inhibition of mitochondria (a source of reactive oxygen species [ROS]), by rotenone [136]. Therefore, repetitive mechanical overload in vitro was shown to cause a significant decrease in mitochondrial SOD concentrations despite increased oxidative damage, which is often observed in chondrocytes from severely arthritic knees [137].

#### 3.7.3. Repurposing of Antidiabetic Agents for the Treatment of Osteoarthritis

Human and animal studies have shown that nearly all antidiabetic drugs are effective in treating OA. For example, antidiabetic treatment with insulin or pioglitazone effectively prevented severe experimental OA in rodents with diabetes compared to those without diabetes in a diabetic rodent model [138]. Some human studies have shown protective effects of antidiabetic medications in knee OA; these drugs were shown to reduce knee OA progression [139]. Additionally, antidiabetic drug repaglinide used in combination with oral NSAIDs is known to alter the pharmacokinetics of the latter in patients with OA [140]. In vitro studies have shown that exenatide, a specific glucagon-like peptide agonist, prevents degradation of type II collagen and aggrecan in articular cartilage by inhibiting MMPs and ADAMTS. It was also shown to reduce oxidative stress and inhibit p38-dependent activation of nuclear factor kappa-light-chain-enhancer of activated B cells (NFkB) [141]. Saxagliptin, a novel dipeptidyl peptidase IV (DPP-4) inhibitor, suppresses the degradation of type II collagen in primary human chondrocytes by decreasing MMP, ADAMTS, and ROS production and increasing glutathione levels mediated by p38/NFkB pathway [142]. Metformin, the most commonly used antidiabetic drug, reduced inflammation, decreased the number of T helper 17 (Th17) cells, and increased the number of regulatory T (Treg) cells in a mouse model of collagen-induced arthritis [143]. Therefore, it was reasonable to conclude that antidiabetic medications attenuate the deleterious effects of DM in patients with OA, and this action halts disease progression [144].

Notably, studies have proved that metformin also effectively treats pancreatic cancer [145], colorectal cancer [146], all types of breast cancer [147], and benign prostate hyperplasia [148]. Moreover, metformin inhibits hepatitis C virus replication in Huh7 cells [149] and counteracts the effects of *Plasmodium* (the parasite causing malaria) [150] as well as other intracellular pathogens such as *Mycobacterium tuberculosis* and *Legionella pneumoniae* [151, 152]. Metformin might also be useful in patients with coronavirus (COVID19) disease, particularly in those with obesity and diabetes [153]. The broad specificity of antidiabetic drugs such as metformin is attributable to their capacity to inhibit hepatic gluconeogenesis and lower blood glucose concentration, improve insulin sensitivity in muscle, and lower plasma concentrations of free fatty acids [154]. The mechanism of action of metformin includes activation of AMPK expression [155] and inhibition of mTOR, which is upregulated in OA [156] either directly by AMPK stimulation or in AMPK-independent process mediated by upregulation of the regulated in development and DNA damage responses 1 (REDD1) [157]. It also affects the mitochondrial oxidative phosphorylation system by targeting Complex I of the mitochondrial respiratory chain [158]. Therefore, the efficacy of antidiabetic medications for the treatment of various disorders including OA could be attributed to their ability to target specific mitochondrial components such as OXPHOS complexes, oxygen consumption, mitochondrial replication, and biogenesis as well as enzymatic activity [158] primarily, via a metformin-mediated decrease in ATP production by the inhibition of the mitochondrial electron transport Complex I [159] resulting in improved insulin sensitivity.

#### 3.7.4. Calorie Restriction for Amelioration and Prevention of Osteoarthritis

OA is associated with obesity and diabetes. Moreover, the maintenance of healthy body weight is essential for the prevention and amelioration of several chronic diseases [160]. Therefore, calorie restriction (CR) could serve as a useful therapeutic alternative in patients with OA. CR represents a nongenetic and nonpharmacological intervention to improve patients' life- and healthspan, primarily by focusing on weight loss [161]. Dietary restrictions mainly act by inhibiting key nutrient sensing and proinflammatory pathways, activating molecular pathways stimulating proteostasis, and enabling genome stability, stress resistance, and stem cell function [162]. The most important result of weight loss is a significant reduction in IR [163] because visceral obesity shows a strong inverse correlation with insulin sensitivity [164]. For example, studies performed in patients with T2D have shown that although the effect of CR on fasting glucose concentrations was variable, fasting insulin concentrations were reduced by 10–40% after 6–12 weeks of CR [165]. Animal studies (in Hartley guinea pigs) have also shown that CR with a regular chow diet reduced knee OA and decreased local and systemic inflammation [166]. However, lifelong CR was not associated with changes in OA incidence of joint synovitis in aged mice, indicating that the reduction of calorie intake is not sufficient to prevent age-related OA [167]. This observation is supported by the results of numerous studies that prove that CR is more effective when combined with physical activity [161].

#### 3.7.5. Activation of Energy Expenditure by Physical Training

Muscle tissue is the first tissue to become insulin-resistant; therefore, changes in plasma glucose and insulin are primarily determined by the muscle IR state rather than insulin insensitivity in adipose or hepatic tissues [168]. Exercise has been demonstrated to activate the mobilization of progenitor cells from bone marrow, neural, and cardiac tissues, which are involved in the recovery of corresponding organs [169]. Therefore, exercise is considered an important physiological regulator of energy utilization as it is capable of increasing the energy metabolism of the whole body by up to 20-fold [170]. The mitochondrial biogenesis induced by exercise also occurs in tissues other than skeletal muscle, including the brain, adipose tissue, and kidney, providing evidence that exercise increases metabolic demand in these tissues and/or stimulates interorgan crosstalk [169].

The high-intensity exercise involves increased glucose oxidation followed by the upregulation of oxidative phosphorylation and anaerobic glycolysis. These processes occur independently of insulin, as the concentration of insulin is very low during exercise [171]. Indeed, insulin and exercise can independently increase glucose uptake as contraction stimulates GLUT-4 translocation through different molecular mechanisms. In muscle cell plasma membranes, the GLUT-4 concentration is regulated by the relative efficacy of exocytosis, which is increased by insulin, and endocytosis, which is reduced by insulin. Muscle contraction increases the rate of exocytosis, while AMPK activation decreases the rate of endocytosis. In addition, the existence of two putative intracellular pools of GLUT-4 was suggested, one of which is recruited by insulin while the other is activated by contractions [172].

During prolonged exercise, FAs start to be used as an energy source, promoting lipogenesis and increasing OXPHOS and protein synthesis associated with increased mitochondrial biogenesis [173]. These effects improve insulin sensitivity [174]. However, in the absence of physical activity, the TCA cycle remained transcriptionally inactivated and was inhibited by ROS and insufficient levels of intermediates [175].

Elevated insulin-independent glucose uptake during exercise was reversed at 2-3 h after exercise. However, increased whole-body insulin sensitivity can persist up to 24–48 h [176]. After acute exercise, insulin-resistant humans and rats can reach glucose uptake values similar to healthy but unexercised controls [177]. In OA animal studies, exercise improved glucose tolerance and inhibited the expression of proinflammatory cytokines while upregulating the expression of the anti-inflammatory cytokine IL-10 [178]. In patients with knee OA, increases in intra-articular and perisynovial concentrations of anti-inflammatory IL-10 accompanied by decreased levels of TNF receptor 1 and increased physical function were also observed in response to exercise [179].

Recent studies have shown that AMPK activators can serve as exercise mimetics [180]. However, both direct and indirect AMPK activators can target one or several cellular pathways, while IR can be caused by numerous mechanisms. Therefore, so far, only exercise can alter homeostasis in all affected cell types [169] and improve insulin sensitivity, while any pharmacological strategy aiming to mimic exercise would need to induce increased energy expenditure similar to that in insulin-sensitive tissues [181].

Therefore, physical training targeting the activation of energy expenditure in the insulin-resistant tissues is viewed as a treatment option for OA and T2D patients.

## 4. Conclusions

Insulin sensitivity is a metabolic tool for discriminating between nutritional challenges and body defence against pathogenic agents and is regulated by reprogramming cellular energy metabolism in both peripheral tissues and immune cells. Therefore, the metabolic changes associated with acute and chronic inflammation might play an adaptive role in restoring the energy homeostasis of the whole body. Insulin resistance is observed in a wide range of different metabolic diseases, including T2D and OA, indicating the inability of the body to overcome energy-related disturbances. Hence, reprogramming energy metabolism could be considered a unifying concept to better understand the pathophysiological mechanisms of chronic inflammatory metabolic diseases such as T2D and OA and to provide new approaches for treatment. As a crucial regulator of energy metabolism at both the cellular and whole-body levels, AMPK controls an integrated signalling network responsible for metabolic adaptation. In addition, AMPK can serve as an indicator of cellular energy imbalance as it is regulated by the AMP/ATP ratio. The energy gridlock indicated by the downregulation of AMPK in insulin-resistant tissues might represent a new therapeutic target in the treatment of T2D and OA. As presently available AMPK activator mimetics fail to resolve tissue energy congestion, exercise could be considered an important physiological regulator of energy utilization, increasing the energy expenditure of the whole body, including AMPK upregulation in different tissues. Therefore, long-term physical training accompanied by calorie restriction and antidiabetic agent treatment could be viewed as a current therapeutic solution for the management of metabolic disorders such as OA.
